# The structural origin of anomalous properties of liquid water

**DOI:** 10.1038/ncomms9998

**Published:** 2015-12-08

**Authors:** Anders Nilsson, Lars G. M. Pettersson

**Affiliations:** 1 Department of Physics, AlbaNova University Center, Stockholm University, SE-10691 Stockholm, Sweden

## Abstract

Water is unique in its number of unusual, often called anomalous, properties. When hot it is a normal simple liquid; however, close to ambient temperatures properties, such as the compressibility, begin to deviate and do so increasingly on further cooling. Clearly, these emerging properties are connected to its ability to form up to four well-defined hydrogen bonds allowing for different local structural arrangements. A wealth of new data from various experiments and simulations has recently become available. When taken together they point to a heterogeneous picture with fluctuations between two classes of local structural environments developing on temperature-dependent length scales.

## Abstract (web summary)

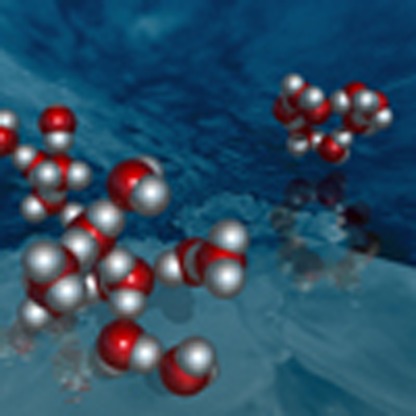
Water is the most common liquid in nature, with unusual properties that could be linked to the peculiar hydrogen-bonding network holding the molecules together. Here, Nilsson and Pettersson review recent progress in searching the connections between local configurations and thermodynamic responses of water.

Water is the most important liquid for our existence and plays an essential role in physics, chemistry, biology and geoscience. What makes water unique is not only its importance but also the anomalous behaviour of many of its macroscopic properties. The ability to form up to four hydrogen bonds (H-bonds), in addition to the non-directional interactions seen in simple liquids, leads to many unusual properties such as increased density on melting, decreased viscosity under pressure, density maximum at 4 °C, high surface tension and many more (see, for example, http://www.lsbu.ac.uk/water/index.html). If water would not behave in this unusual way it is most questionable if life could have developed on planet Earth^1^.

Figure 1 shows the temperature dependence of the isobaric heat capacity (*C*_P_) and the isothermal compressibility (*κ*_T_) that initially decrease with decreasing temperature but, just above the ambient temperature regime, there is a deviation and both the heat capacity and compressibility begin to increase on further cooling, resulting in a minimum at 308 K (35 °C) and 319 K (46 °C), respectively^2^,^3^,^4^,^5^,^6^. These properties are related to fluctuations in the liquid where *κ*_T_ derives from volume, or equivalently density, fluctuations, whereas *C*_P_ is related to entropy fluctuations^2^,^7^. Similarly, the density (*ρ*) of a simple liquid increases with decreasing temperature and this is true also for water when it is hot. However, as it enters the ambient regime, the rate of increase decreases and a maximum in the density is seen at 277 K (4 °C) after which the density instead decreases on further cooling^8^,^9^,^10^. The density variation can be derived from the thermal expansion coefficient (*α*_P_), dependent on the cross-correlation between fluctuations in density and entropy, which thus becomes negative for water below the density maximum^2^,^7^. As these thermodynamic properties measure the response to external perturbations, such as temperature or pressure, they are referred to as thermodynamic response functions^11^. Their unusual behaviour for water is denoted anomalous where the anomaly becomes much stronger on cooling. Indeed, when fitted to a power law each of these properties seems to diverge towards a temperature of 228 K (ref. 4).

To gain fundamental understanding of the origin of these anomalies we need to address the instantaneous local structure of the liquid at various thermodynamic state points and establish how this structure couples to the dynamics of the molecular motion. The starting question is: on a larger scale liquid water is homogeneous, but is it also locally homogeneous or can it be heterogeneous (see Box 1)? Not in terms of a static structural picture, but induced by fluctuations on some length and time scales between specific classes of local structures. Many different plausible explanations exist for the unusual properties of water where maybe both homogeneous and heterogeneous models could be viable and sophisticated structural and dynamical experimental data are needed to determine their validity.

The other major question is: how large are distortions in the H-bonding network? In a locally homogeneous model the distortions should be around a near-tetrahedral H-bond arrangement, whereas in a heterogeneous model there would be distortions within each class of configurations in addition to the distinction in local structure between the classes. The more popular heterogeneous models build on fluctuations between two main classes of contrasting structures with notations such as tetrahedral and distorted^12^, symmetrical and asymmetrical^13^,^14^, locally favoured and normal^15^, and low-density liquid (LDL) and high-density liquid (HDL)^7^,^16^,^17^,^18^ to mention the most recent proposals; these refer to the same two general structural classes where the notation reflects which specific properties are brought forward by the various experimental^7^,^11^,^12^,^16^,^17^,^18^ and modelling^13^,^14^,^15^ techniques.

Another much debated question is whether such heterogeneous fluctuating structures could develop into metastable macroscopic phases on extreme supercooling^19^,^20^. Would this also lead to the existence of a second critical point^19^? A liquid–liquid critical point (LLCP) has never been observed for a one-component fluid, but the apparent power-law divergence of compressibility and heat capacity on supercooling water has been proposed to be an indication of an LLCP^19^.

Here we will review developments in the last few years around the question whether pure bulk water is structurally heterogeneous or homogeneous and how the answer may relate to its unique anomalous properties. At the end we propose a unified picture that can explain many observations in both the ambient and supercooled regimes.

## Electronic structure and vibrational spectroscopy

Transitions between different electronic or vibrational states give specific spectral signatures that can shed light on possible unique structural environments. The goal of this section is to establish trends in these spectral features based on experimental observations of their temperature dependence and perturbations due to added salt.

### Local structure probed by X-ray spectroscopies

Here we first review electronic spectroscopies involving core level excitations to and from the valence levels. Figure 2a shows the temperature dependence in X-ray absorption spectroscopy (XAS), which probes the unoccupied states and was recently obtained also for slightly supercooled water^21^. There is general agreement that the pre- (535 eV) and main-edge peaks (537–538 eV) fingerprint distorted H-bonds, whereas the post edge (540–541 eV) is associated with strong H-bonds and is further enhanced for tetrahedral H-bond structures^13^,^22^,^23^,^24^,^25^. This is consistent with the temperature dependence where the post edge, which dominates in hexagonal ice, loses intensity to the pre-edge and main edge on heating as seen in Fig. 2a (refs 21, 22). The spectral features do not undergo any major shift or broaden with increasing temperature, but instead there are changes in intensity at an almost fixed energy. It has also recently been shown that the main-edge intensity becomes enhanced on formation of high-density forms of ice such as high-density amorphous (HDA) ice^26^ and various crystalline high-pressure forms such as ice II, VI, VII and VIII^27^. The effect on adding NaCl salt is a decrease of the post-edge spectral feature and increase in the pre-edge and main-edge features similar to the changes with temperature^22^,^28^; adding NaCl salt has furthermore been shown to affect water structure similar to increased pressure, that is, reducing the tetrahedrality^29^ (see next section). Figure 2b shows that the occupied non-bonding 1*b*_1_ lone-pair in X-ray emission spectroscopy (XES) is split into two components where the intensities show dependence on temperature^30^,^31^ and on adding NaCl^32^. Here, again there is no broadening or major shift in the two spectral components, only intensity changes. The origin of the split has been discussed as due to two different instantaneous structural environments giving different electronic emission energies in the spectroscopic process^31^,^33^,^34^ or giving different O–H stretch dynamics in the core ionized intermediate state^35^,^36^. Through resonant excitations corresponding to various parts of the XAS spectrum, a direct connection has been shown between the two spectroscopies where the low-energy 1*b*_1_ corresponds to the post edge, and that at high energy correlates with the pre- and main edges fully consistent with the temperature and NaCl concentration dependences^31^,^33^. Curve fitting of the XAS and XES spectra at ambient temperature gives around 20–30% locally tetrahedral molecules^25^,^31^, which is consistent with estimations in two-state thermodynamic models^15^,^37^.

### Structure and dynamics probed by vibrational spectroscopies

We next describe trends from vibrational spectroscopy in both the OH stretch region (Fig. 2c)^38^,^39^ and in the low-energy vibrational modes (Fig. 2d)^40^. Important information about H-bond dynamics has been obtained using two-dimensional spectroscopy^41^,^42^, but here we discuss linear spectroscopy to connect the trends with those obtained from X-ray spectroscopies. Often the focus has been on the OH (OD) stretch spectrum in HDO (HDO is water with one proton (H) substituted by deuterium (D)) in D_2_O (H_2_O), which effectively decouples the oscillator from the environment and makes the OH (OD) group a local probe of the H-bonding^41^. In this case only a broad spectral feature without fine structure is observed and the temperature dependence shows a redistribution of intensity from one side towards the other, opening for many different interpretations in terms of both homogeneous and heterogeneous distributions^14^,^39^,^41^. However, when the coupling to the surrounding liquid is turned on in neat H_2_O, the spectrum becomes different due to coupling of resonances through H-bonds with neighbouring molecules^41^,^43^,^44^.

The OH stretch vibration in H_2_O has been studied for many years^45^ but recently Sun^38^,^39^ compared measurements over an extended range of temperatures for pure water and a range of concentrations of NaCl solution at fixed temperature and found consistent behaviour of the well-resolved spectral feature on the low-frequency side of the OH spectrum close to the broad spectral distribution in hexagonal ice^46^. We note that this feature shows the same temperature and NaCl concentration dependence^38^,^39^ as the post edge in XAS^22^ and the low-energy component of the split 1*b*_1_ peak in XES. In addition, from the time-resolved optical Kerr effect measurements by Taschin *et al.*^40^, involving low-energy vibrations in the H-bonding network, there are clearly identified signatures of two components in the low-frequency region with the same temperature dependence as the other spectroscopies (Fig. 2d). A further indication of heterogeneities in water is given by recent pump–probe vibrational spectroscopy, which finds a significantly weaker coupling to other chromophores when exciting on the blue side of the spectrum than on the red^47^. Consistent with this weaker interaction, vibrationally resolved XES spectra^48^, using resonant excitation at the pre-edge in XAS, result in an OH-stretch frequency very near that of gas phase water supporting the interpretation of the pre-edge as due to molecules with broken or weakened donated H-bonds^24^,^25^. In this resonantly excited case, only the high-energy spectral component was observed in the 1*b*_1_ valence band region^16^.

### Connecting X-ray and vibrational spectroscopy information

Thus, the X-ray and vibrational spectroscopies show similar trends with spectral features at fixed energy and only changes in intensity with temperature and NaCl concentration. These observations are consistent with two distinct, different structural classes where the population in each varies with temperature and salt concentration. As it is well known that increasing temperature^49^ and NaCl concentration both remove the tetrahedral coordination^29^, it is clear that the increase in the post-edge shoulder, the low energy 1*b*_1_ component, the 3,200 cm^−1^ OH stretch and the 225 cm^−1^ low-energy vibrational regions point to an environment connected to tetrahedral structures. These features are also fully aligned with the corresponding features of hexagonal ice. If the tetrahedral structures with strong H-bonds are collective and involve several other tetrahedral water molecules, as discussed in the next section, it could potentially explain the appearance of the 3,200 cm^−1^ stretch vibration (not seen in HDO diluted water) as due to coupling to neighbouring tetrahedral water molecules^41^.

On the contrary, the pre-edge and main-edge XAS resonances, the high-energy 1*b*_1_ component, the 3,400 cm^−1^ OH stretch and the 180 cm^−1^ low-energy vibrational regions can be related to non-tetrahedral or more distorted structures. These features completely dominate when tetrahedral order has been removed.

### Magnitude of distortions in disordered structures

An important question is how to determine the magnitude of distortions that could give rise to the pre-edge feature in XAS, as it is clearly related to the blue part of the OH stretch spectrum and to the high-energy component in the split 1*b*_1_ peak in XES. One way to investigate such distortions around the first shell is through theoretical energy decomposition of H-bonds in *ab initio* molecular dynamics (MD) simulations, as recently done by Khaliullin and Kühne^13^,^50^. This reveals a significant asymmetry in terms of H-bond strength, both for the two donor and for the two acceptor bonds. Figure 2e shows the asymmetry parameter *Y* for acceptor and donor bonds, where *Y*=0 when the two H-bond strengths are equal and *Y*=1 when the two bonds are extremely asymmetric with one nearly broken and the other very strong^13^. In the simulations an asymmetry of around *Y*=0.5 is predominant, which in terms of geometry corresponds to small deviations in the H-bond length, and thus a predominance of tetrahedral structures. However, there is a fraction of molecules in the upper right corner of Fig. 2e with very high asymmetry that Khaliullin and Kühne demonstrated contribute high pre-edge peak intensity in the computed XAS spectrum of water^25^. In this particular *ab initio* MD simulation related to Fig. 2e, the fraction of highly asymmetrical species was small but the simulation is overstructured compared with experiment^13^,^50^, indicating that in real water this fraction should be significantly larger.

## Simulations and X-ray scattering

The most direct way to obtain structural information in liquids is from the pair-distribution functions (PDFs) that measure correlations at various distances. Let us first visit a simulation using the TIP4P/2005 force field^51^ to inspect how the O–O PDF (Fig. 3a) appears for different structural environments distinguished based on the local structure index (LSI)^52^. What characterizes tetrahedral structures is a well-defined separation of the first shell at 2.8 Å and the second shell at 4.5 Å (high LSI), whereas structures that are less tetrahedral contain interstitials that fill in the region between 3 and 4 Å (low LSI)^53^. The interstitials are typically seen for high-pressure ices due to inwards collapse of the second shell. Very similar results using an LSI classification have been obtained by Car and colleagues^54^ based on advanced *ab initio* MD simulation techniques. From neutron-scattering experiments similar O–O PDFs have been derived by extracting data at various temperatures and pressures, and extrapolating to pure water phases, to correspond to extremely tetrahedral environments, or with a high degree of interstitials that were denoted LDL and HDL phases, respectively^17^,^18^. This notation comes from that the interstitial structures typically appear when applying pressure to generate a high-density phase in either water or ice. The same occurs, as discussed in the previous section, when NaCl is added to water where the 4.5 Å correlation disappears and instead the number of interstitials increases. The tetrahedral structures, on the other hand, create more open space and are therefore denoted a low-density phase. We note that for the low-LSI PDF there is only a first shell peak at around 2.8 Å but the rest is rather unstructured. This shows that the interstitial-related environment is much more disordered, but the first peak is also much lower and broader, indicating that also the first coordination shell is much more distorted, which is in line with the discussion of the distorted structures in the preceding section.

### Pair-distribution functions

The ability to perform X-ray-scattering experiments on water, both in the low-11,16,55 and high-*Q*^49^,^56^ momentum transfer regions, has recently taken a major leap forward, providing further insights on the heterogeneity of the liquid. In recent works, Skinner *et al.*^49^,^56^ used extremely high-energy X-rays allowing for measuring a large *Q*-range on a single detector that allows extracting accurate O–O PDFs. They report a decreasing height of the first O–O correlation with increasing temperature combined with an isosbestic point in the coordination number at 3.3 Å, meaning that the number of molecules in the first shell with this cutoff remains constant between 254 and 342 K (ref. 49). This shows that as the first shell peak height decreases, correlations are shifted from 2.8 to the 3.0–3.3 Å region, meaning that interstitials build up not only from the collapse of the second shell but also from distortions of the first shell.

**Figure 1:**
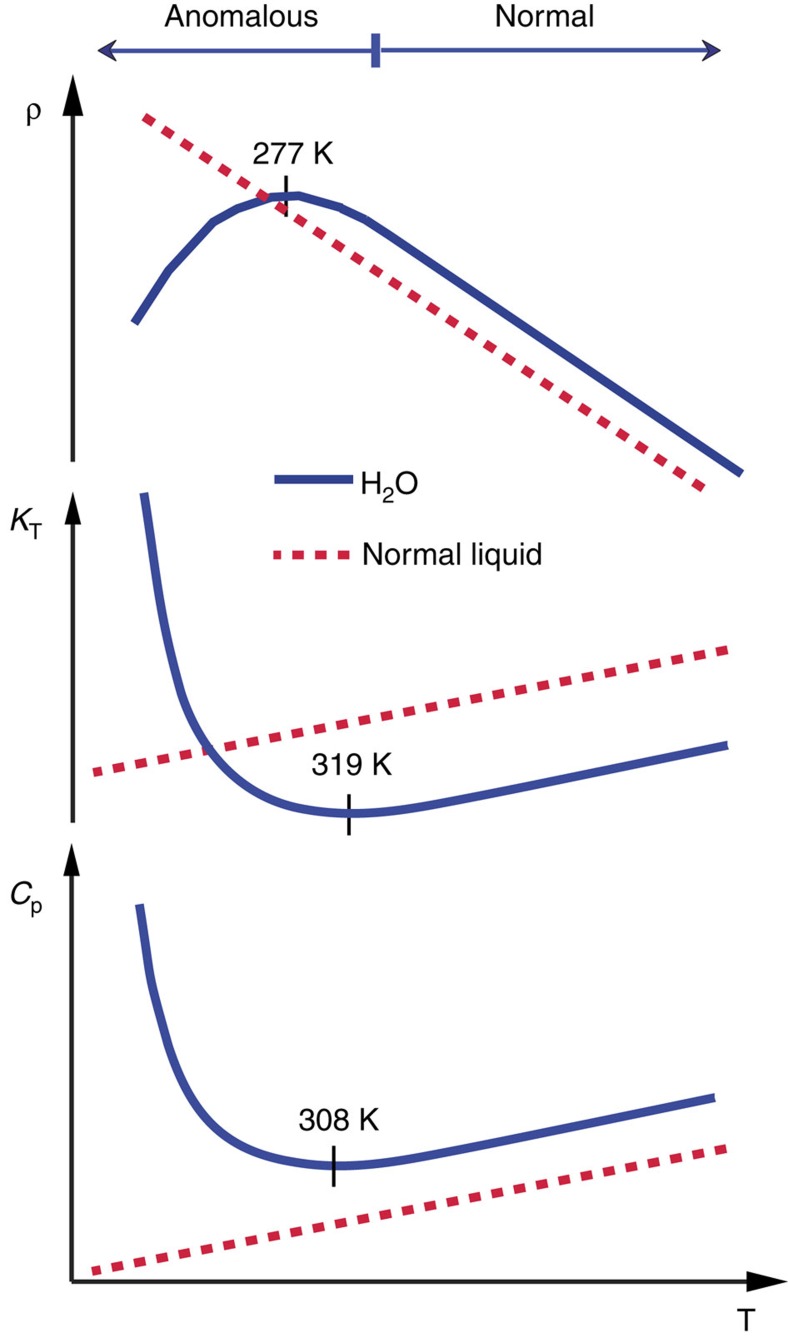
Thermodynamic properties. Comparison of the density (ρ), isothermal compressibility (*κ*_T_) and heat capacity (*C*_P_) for H_2_O water (full line) with that of typical liquids (dashed) showing the onset of anomalous behaviour already at ambient temperatures and pressure.

**Figure 2:**
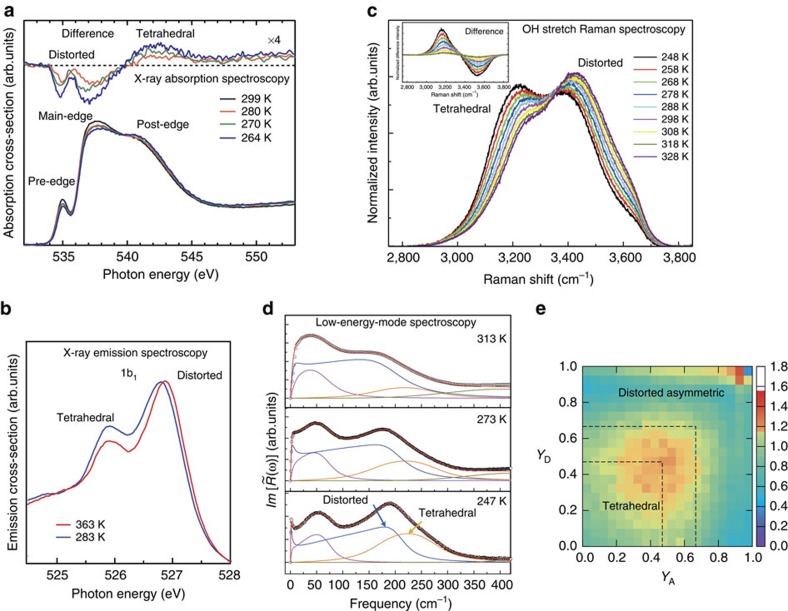
Local structure probes. (**a**) XAS spectra of liquid water measured in transmission as function of temperature. The top section shows the difference in absorption cross-section compared with the spectrum measured at 299 K. The difference in absorption cross-section was magnified by a factor of 4, to highlight the spectral changes; the dotted black line denotes no difference. (Adapted from ref. 21). (**b**) XES spectra in the lone pair 1*b*_1_ region of D_2_O liquid water measured at 283 and 363 K. The heavier isotope is used to minimize core hole-induced dynamics. (Adapted from ref. 16). (**c**) OH-stretch Raman spectrum as a function of temperature with an inset showing the difference spectra (Adapted from refs 38, 39). (**d**) The Fourier transform of the time-dependent optical Kerr response as a function of temperature. This is directly related to the frequency-dependent spectrum measured in the dynamic light scattering. The contributions of the three slave correlators are reported in the figure as magenta–blue–orange lines. (Adapted from ref. 40). In each figure the dominant spectral contributions interpreted as due to H-bond distorted or tetrahedral character are indicated, showing consistent trends with temperature, NaCl concentration and O–O pair-distribution function (PDF). (**e**) The normalized probability density function of the asymmetry parameters YA (acceptor) and YD (donor). (Adapted from ref. 50).

**Figure 3:**
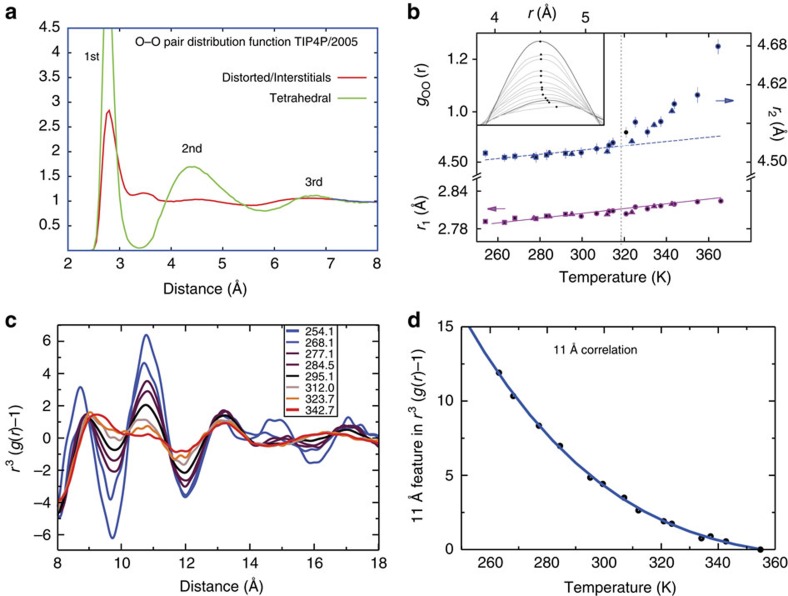
Pair-distribution functions. (**a**) O–O PDF of TIP4P/2005 water at 298 K separated into PDF:s between molecules with LSI value above and below a threshold of 0.05, showing extremes of LDL-like and HDL-like components, respectively, in the simulation. (**b**) Positions of the first (*r*_1_) and second (*r*_2_) peak maxima in the O–O PDF as a function of temperature. Inset shows the details of the second peak. (Adapted with error bars from ref. 49). (**c**) The O–O PDF as function of temperature (K) plotted as 4π*r*^3^(*g*(*r*)−1) to enhance features at long distance (Adapted from ref. 49). (**d**) The temperature dependence of the 11 Å correlation from **c**.

Figure 3b shows the temperature dependence of the positions of the first two shells as extracted from the O–O PDFs^49^. As expected, the radial distance of the first shell increases linearly with increasing temperature, as the liquid expands with increasing temperature. The second shell follows the same trend with a similar increase in distance up to a temperature around 320 K, but then there is a dramatic increase and the second peak in the PDF becomes more undefined. This change occurs around the same temperature as the minimum in the isothermal compressibility, shown in Fig. 1. There are some even more striking changes in the liquid if we look at shells at longer distances. Figure 3c shows that the new high-quality data clearly resolve even up to eight shells with the farthest at around 17 Å and they show very distinct differences in their temperature dependence. Previous work has indicated that the feature around 11 Å can be affiliated with local regions of tetrahedral structures^53^,^57^. Figure 3d shows the temperature dependence of the peak height of the correlation at 11 Å. At high temperatures the height is near zero but then the 11 Å correlation starts to increase around 340–320 K as the liquid is cooled. This shows that it is only around the compressibility minimum that H-bonding becomes decisive and collective tetrahedral fluctuations begin to result in well-defined regions that have a radial extent around 11 Å. This explains the observation in Fig. 3b in terms of the second shell, as it is at 320 K that the tetrahedral structures become well defined as collective regions, whereas at higher temperatures these are much less well defined.

### Small-angle X-ray scattering

Such a spatial extent of heterogeneities has earlier been proposed based on small-angle X-ray scattering measurements of water where a temperature-dependent enhancement of the structure factor at low momentum transfer *Q* has been reported^16^,^53^,^55^. Such an enhancement is typically seen for nanoparticles and aggregates in solutions allowing determination of sizes and shapes, but this should also be applicable to density fluctuations, as these are significantly slower than the attosecond X-ray photon-scattering time and an average spatial extent of ∼10 Å at ambient conditions was proposed based on the measured correlation length ∼3.1 Å (ref. 16). This interpretation was criticized by Clark *et al.*^58^,^59^ and Soper *et al.*^60^ who instead proposed that the data were fully consistent with a homogeneous description. More insight has recently been obtained through work by Overduin and Patey^57^ and Wikfeldt *et al.*^61^,^62^, where TIP4P/2005 force-field simulations show trends qualitatively consistent with the experimental data and provide information regarding the spatial distribution and extent of the structures in the liquid consistent with the originally proposed spatial extent^16^. It is noteworthy that the concept of correlation length is related to the asymptotic decay of the correlation function and the size range of regions is nearly a factor 4 larger^11^, which is consistent with the development of the experimentally observed 11 Å correlation. What is interesting is that the enhancement at low *Q* appears around 320 K on cooling, which correlates well with the changes in Fig. 3d. This is even more clearly seen in the comparison of the total and normal contributions to the isothermal compressibility as a function of temperature in the work by Overduin and Patey^57^. At high temperatures the normal component is nearly equal to the total and both decrease with decreasing temperature as expected for a simple liquid (Fig. 1a). However, close to the minimum around 320 K the normal component begins to deviate from the total with another anomalous component becoming increasingly significant as the liquid is cooled further^57^.

### Anomalous properties and long-range correlations

All these data indicate that, at the compressibility minimum around 320 K, water becomes anomalous not only in terms of the thermodynamic response functions but also in the structure with local regions of tetrahedral structures ∼11 Å in average radial extent appearing as fluctuations in the distorted/interstitial structurally dominated liquid; these furthermore grow to even larger sizes on further cooling. Here it is important to realize that many-body effects are known to give rise to H-bond cooperativity effects where water molecules prefer to bind with molecules that are in a similar surrounding, leading to local regions^57^,^63^. At temperatures above 320 K, where structural fluctuations are rare and water behaves as a simple liquid, the structure is more homogeneous.

## Supercooled water

In the supercooled regime the nomenclature HDL and LDL has some historical connection with the LLCP hypothesis that water could exist as HDL and LDL macroscopic phases^19^. This links strongly to the glassy state of water in terms of HDA and low-density amorphous ices, and the transformation between them^7^,^64^. As the structural characteristics of LDL and HDL phases are related to, respectively, a well-defined peak at 4.5 Å and the presence of interstitials around 3–4 Å (ref. 17), we will, for simplicity, use this notation also for local structures appearing as fluctuations with O–O PDFs similar to those of the macroscopic phases, although the local density as such is not a well-defined property.

### Validity of a two-state model

The very recent works of Holten and Anisimov^37^, and Russo and Tanaka^15^ building complete thermodynamic equations-of-state for water show the validity of a two-state picture with fluctuations between HDL and LDL local structures appearing in the transition between the two phases. These equations-of-state reproduce the temperature dependence of the thermodynamic response functions shown in Fig. 1 and indicate that an LLCP at positive pressure is necessary to describe the data. Here we will use some of this modelling together with new experimental data in the deeply supercooled regime to demonstrate consistency with the LLCP and discuss trends with respect to simulations.

### Two liquid phases

**Figure 4:**
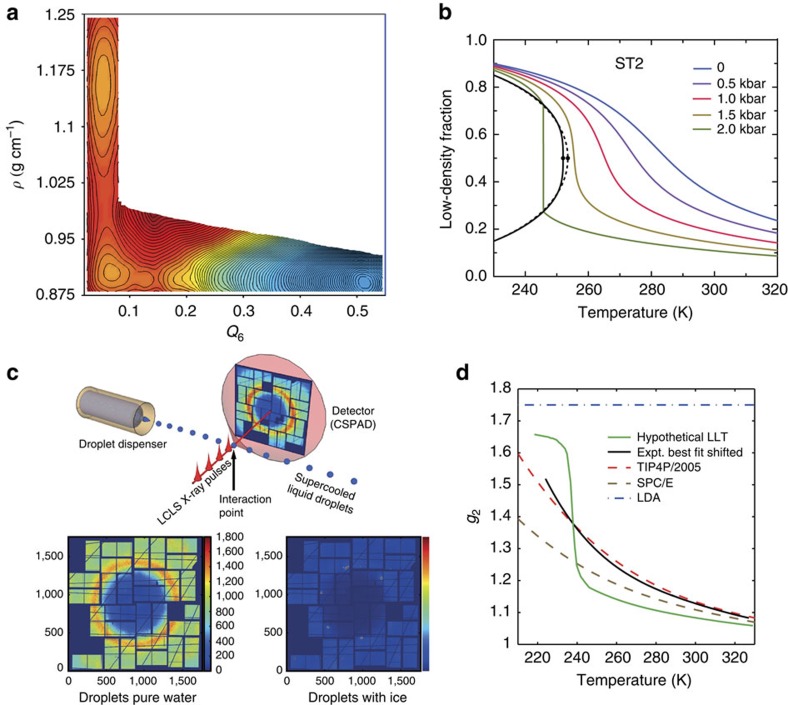
Supercooled water. (**a**) An orthographic projection of the reversible free-energy surface described by density and the crystalline order parameter, *Q*_6_, for ST2 water at a point of liquid–liquid coexistence (228.6 K and 2.4 kbar). (Adapted from ref. 20). (**b**) Low-density fraction from the predictions from the cross-over equation of state extrapolated from the ST2 model as a function of temperature at various pressures. (Adapted from ref. 70). (**c**) A train of droplets flows in vacuum perpendicular to ∼50 fs long X-ray pulses. A coherent scattering pattern from a water droplet was recorded when a single droplet was in the interaction region at the time of arrival of a single X-ray pulse. Each diffraction pattern is classified either as a water shot exclusively containing pure liquid scattering characterized by a diffuse water ring or as an ice shot characterized by intense and discrete Bragg peaks superposed on the water-scattering ring. (Adapted from ref. 71). (**d**) Magnitude of the second *g*(*r*) peak, *g*_*2*_, as function of temperature for SPC/E and TIP4P/2005 simulations, hypothetical LLT and a curve fitted to the experimental data^71^ but shifted to 5 K lower temperatures to cross the TIP4P/2005 simulation curve at its Widom line. LDA marks g_2_ for low-density amorphous ice.

There has recently been an intense debate regarding the validity of the two-state model with a liquid–liquid transition (LLT) and a LLCP at positive pressure^20^,^65^,^66^,^67^. This was based on simulations, as no experiments can yet sample this region of the metastable water phase diagram due to rapid ice crystallization. Figure 4a shows the free-energy surface from Palmer *et al.*^20^ using the ST2 water model^68^ for which coexisting metastable basins of HDL and LDL are found at the thermodynamic conditions corresponding to an LLT. There is a free-energy barrier between the metastable LDL and the thermodynamically stable cubic ice phase, indicating a LLT and LLCP in the ST2 model. By modifying the H-bond angular flexibility in ST2 it is furthermore possible to shift the LLCP to become located in the stable part of the phase diagram with respect to ice formation, confirming the validity of a LLCP in atomistic simulations^69^. Based on the ST2 model, Holten *et al.*^70^ estimated the fraction of LDL local structures as a function of temperature. The resulting curves indicate the existence of an LLT as shown in Fig. 4b. The derivative becomes infinite at the LLT and LLCP, and becomes smaller as the pressure is lowered into the one-phase region where the change in population very far from the LLCP approaches a linear relationship. This clearly demonstrates that the fluctuations between LDL and HDL (see Box 1) extend far out from the critical point. Similar work by Russo and Tanaka^15^ using the TIP4P/2005 force field and an order parameter to find the fraction of the LDL component (there denoted locally favoured structure) showed almost identical trends as for the ST2 model with only a shift in temperature and pressure. Analysis of the TIP4P/2005 data shows that the derivative becomes infinite at a point that seems to indicate the existence of LLT and LLCP^15^ but it remains to be determined whether the LLCP is real or only virtual^37^ in the TIP4P/2005 model.

### Probing water in ‘No-Man's Land'

It has been difficult to experimentally probe water deep in the supercooled regime due to rapid ice crystallization. Recently Sellberg *et al.*^71^ conducted an experiment at the Linac Coherent Light Source X-ray laser, where they could very rapidly cool micron-size water droplets and probe the liquid structure with X-ray scattering as shown in Fig. 4c. The lowest temperature that was achieved while maintaining the liquid state was 227 K, which is 5 K below the previous limit of homogeneous ice nucleation^72^ defining the upper onset of what has become denoted ‘no-man's land'. Figure 4d shows the development of the tetrahedrality of the liquid, based on the height (*g*_2_) of the second O–O PDF shell at 4.5 Å, as function of temperature with an accelerated change towards a tetrahedral or LDL dominated liquid at the lowest temperatures. The change in the temperature-dependent *g*_2_ slope is larger and occurs at slightly higher temperatures than the change in TIP4P/2005 water and is much larger than for the SPC/E model.

As it is the LDL component that gives the *g*_2_ parameter as the excess above one in the second shell (see Fig. 3a where HDL is an almost flat line), they are directly related. The TIP4P/2005 model gives a similar trend as ST2 in Fig. 4b with the slope of the change becoming infinite at the LLCP at 1,350 bar^15^. In the experiment we would expect a near discontinuity in *g*_2_ (as indicated with the schematic LLT line in Fig. 4d) if the approach were to an LLT or if an LLCP were located at negative^73^ or ambient pressure. It is noteworthy also from Fig. 4b that in this case the LDL fraction would remain nearly constant coming from high temperatures until very close to the discontinuity and thus also *g*_2_ versus temperature would remain constant. In contrast, the measured *g*_2_ in Fig. 4d exhibits at near-ambient pressure a significant and increasing slope more reminiscent of going through the one-phase region in Fig. 4b at pressures lower than the critical. Noting that the measured change becomes more accelerated at low temperatures than in the TIP4P/2005 simulation, we expect the LLCP of real water, if it exists, to be at much lower pressure than in the simulation. From this we can conclude that a rough estimate for the LLCP should lie somewhere between ambient pressure and the TIP4P/2005 LLCP type of behaviour, maybe in the range 500–1,500 bar. This would be within the range of estimates based on measurements on emulsified water by Mishima^74^ and decompression-induced melting of ice IV^75^ but new experiments are needed to determine whether an LLCP really exists or whether it is only virtual. In the latter case it would cause fluctuations as if there would be an LLCP but with ice crystallization occurring too rapidly for criticality to fully develop^76^.

## Proposed unified picture

**Figure 5:**
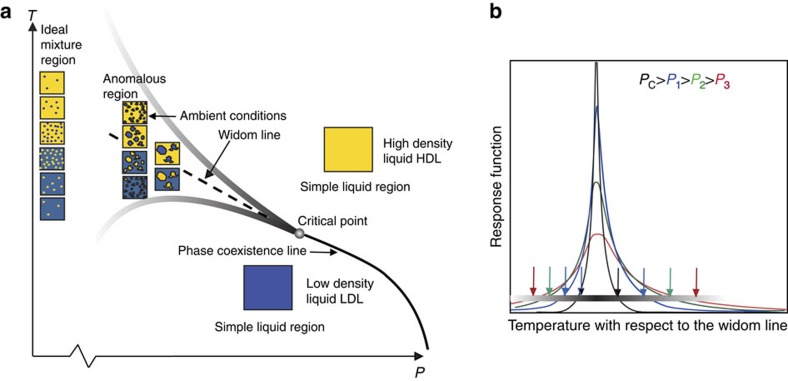
Unified picture. (**a**) Schematic picture of a hypothetical phase diagram of liquid water showing the liquid–liquid coexistence line between LDL and HDL in terms of simple liquid regions, the critical point (real or virtual), the Widom line in the one-phase region and fluctuations on different length scales emanating from the critical point giving rise to local spatially separated regions in the anomalous region. The shaded lines indicate how far in temperature the fluctuations extend at the various pressures defining the anomalous region. (**b**) Schematic diagram of the temperature dependence with respect to the Widom line of a thermodynamic response function, such as the isothermal compressibility (*κ*_T_) or heat capacity (*C*_P_), at various pressures but below the pressure of a critical point. Entering into the anomalous region (shaded line in **a**) can be defined as the temperature when the absolute value of the slope has increased beyond some pre-defined value indicated by arrows.

Here we want to use the latest developments to propose a simple interpretation giving what we consider a more unified qualitative picture in Fig. 5a of water incorporating a broad temperature range and extending the discussion from the supercooled regime also to ambient temperatures, as the anomalies persist up to ∼320 K.

There could exist two separate liquid phases, HDL and LDL, with a coexistence line in the *P*–*T* diagram deep in the supercooled regime and at elevated pressure^7^,^19^. This LLT line ends with decreasing pressure in an LLCP and its extension into the one-phase region corresponds to the Widom line^77^. At the Widom line the density fluctuations would reach a maximum consistent with equal population of molecules in HDL and LDL^62^. It is worth noting that HDL is on the ambient-temperature side of the Widom line, whereas LDL is on the low-temperature side. This explains why the high-density, H-bond-distorted species dominate at ambient conditions. The origin of the anomalous properties of water is the increase in structural fluctuations, as water is cooled down and approaches the Widom line, leading to fluctuations into tetrahedral patches growing in size as directional H-bonding becomes relatively more dominant^16^,^78^. Figure 5b schematically illustrates the temperature dependence of a thermodynamic response function (*κ*_T_ or *C*_P_) at various pressures relative to the Widom line. The response function shows a maximum at the Widom line with the height and width dependent on the distance to the critical point. The fluctuating funnel-like anomalous region (Fig. 5a) around the Widom line becomes wider the further away from the LLCP we are in pressure. The closer we are, the narrower the region becomes; however, in this region the anomalous behaviour becomes more strongly enhanced, leading to steeper rise in the response functions^79^.

We can relate to the fundamental discussion about a heterogeneous contra homogeneous distribution of structures (Box 1) by inspecting more closely the funnel-like anomalous region beyond the critical point. Outside we have the macroscopic phases of HDL and LDL, each a simple liquid that would correspond to the homogeneous description with thermal fluctuations and distortions around only one class of structural motifs. Changes in temperature do not affect the populations consistent with Fig. 4b, where at 2 kbar and above 260 K the tetrahedral LDL fraction is low and near constant. Inside the anomalous region we are in the region of heterogeneous fluctuations; however, as we get further away from the LLCP the size of the fluctuations becomes smaller. Eventually, we will approach a limit where the sizes reach molecular dimensions as in an ideal mixture with many intermediate structures resulting in an almost flat line in the populations as in the top figure of Box 1. This would correspond to an almost linear dependence in the tetrahedral LDL population with temperature as in the 0 bar case in Fig. 4a or further extrapolated to negative pressures. As MD force-field simulations give an LLCP at higher pressures than what is discussed here for real water, the resulting structures at ambient pressure would be closer to the ideal mixture limit. We should also note that this is a simplified picture and the boundary at the funnel-like anomalous region is not sharp, and there will be an extension of small contributions of LDL/HDL local structures but with more of an ideal mixture-like dispersion into the simple liquid regions. This is why tetrahedral structures are seen in the spectroscopic data also at temperatures above the here-indicated anomalous region but without resulting in spatially separated regions.

What is unique with water is that at ambient pressure the location of the LLCP is such that the anomalous region with fluctuations extends up to around 320 K (47 °C). This means that water is anomalous at temperatures where life is sustained and where most processes of importance in nature and to our society occur. It would be interesting in the future to gain insight into whether this is a pure coincidence or has significant implications for understanding biology.

## Future perspective

Coming back to Box 1, a burning question is to determine the degree of heterogeneity in water and the sharpness of the boundaries between the fluctuating regions. This will rely partly on theoretical developments to describe the observed spectroscopic information. Would a large boundary region with more average structure between the two regions show a clear spectral contribution or would it correspond to a projection onto both spectral components? We note here that, in the inherent structure (quenched to 0 K) in simulations, the distribution of the LSI order parameter is highly bimodal with a minimum between HDL- and LDL-like character^54^,^62^,^80^; however, the distributions are not completely separated, indicating molecules in environments of mixed character. In the real structure in simulations (including temperature) the distribution is on the other hand continuous, indicating a more homogeneous liquid with more smeared-out heterogeneities (moving more towards the right in Box 1). It will be important to further investigate the difference in inherent and real structure using models that better include cooperativity effects and bring the LLCP towards lower pressure than TIP4P/2005 or ST2. Having the LLCP at lower pressure would shift the funnel in Fig. 5a and make the onset of fluctuations at ambient pressure more distinct, which would improve, for example, the MD isothermal compressibility, which rises too slowly^81^ and the *g*_2_ parameter in Fig. 4d.

A challenge that requires more experimental developments is to investigate the ‘no-man's land' region more deeply and determine whether or not an LLCP and an LLT exist and whether there is a free-energy barrier to rapid ice formation. There could be a virtual LLCP defined as a point of instability in the *P*–*T* phase diagram giving rise to fluctuations in its neighbourhood as indicated in Fig. 5a, but where too rapid ice nucleation prevents real criticality in terms of diverging correlation length^76^. Here, the experiments recently demonstrated at Linac Coherent Light Source show an interesting avenue if they can be conducted also under pressure, a most challenging task. Another way is to come up from below ‘no-man's land' starting from the glassy state. It has been seen that starting from low-density amorphous or HDA ice leads to different temperatures of the glass transition^82^. The question is whether there is a real liquid state with translational motion at these low temperatures.

There has been a large interest in recent years in H-bond dynamics through the development of infrared pump–probe experiments and two-dimensional spectroscopies^41^,^42^,^83^,^84^. It is important to understand which part of the dynamics can be related to the anomalous contribution from the HDL–LDL fluctuations that causes the enhancement of *κ*_T_ at low temperatures. These fluctuations are naturally related to more collective motions of many water molecules to form and dissolve local LDL regions, which is beyond the simple H-bond dynamics probed through the local OH stretch mode. There are indeed indications from three-dimensional infrared spectroscopy that subassemblies of water molecules at ambient conditions have different dynamics on timescales longer than 0.5 ps^84^. Related to this, a relaxation time of the order of 1.5 ps has been derived from high-resolution inelastic scattering measurements of ambient water^85^. However, it is not yet known whether this dynamics can be related to the anomalous fluctuations connected to the negative slope of the temperature dependence of *κ*_T_ shown in Fig. 1. In particular, it is expected that the dynamics will change abruptly on passing the Widom line in Fig. 5a where the more rigidly H-bonded structure in an LDL-dominated liquid could slow down the dynamics by several orders of magnitude and maybe give rise to the speculated fragile-to-strong liquid transition^86^, that is, from non-Arrhenius to Arrhenius-like temperature dependence of the viscosity. Here, it is also important to develop techniques that probe the true equilibrium dynamics in a probe–probe scheme instead of pump–probe.

Water represents a severe challenge to theoretical simulations due to the delicate balance between different counteracting interactions of similar magnitude and also to the emergent character of the resulting properties, which requires a large number of molecules to develop. It is clear that highly accurate computational approaches are required to reliably capture this delicate balance, but these need also to be applicable to large-enough systems and long-enough trajectories to allow fluctuations on relevant length and time scales to develop. Classical force-field simulations can easily be applied to very large boxes for rather extended simulations, but with questionable accuracy. Recent force-field developments with parameterization of up to three-body interactions at the CCSD(T) level of quantum chemical accuracy hold promise to remedy this^87^, but whether this approach is sufficient to capture all aspects of the water phase diagram remains to be seen. In another approach, VandeVondele and colleagues^88^ have exploited algorithmic and computer developments to perform Monte Carlo simulations of 64 water molecules at the fully *ab initio* periodic quantum chemical MP2 level; however, considering the experimental observation of an eighth radial coordination shell at ∼17 Å, significantly larger simulation boxes will be necessary, which will be a challenge to this approach. Simulations based on Density Functional Theory (DFT)^89^,^90^ scale better with size of the system but a box size of 30–40 Å still represents a severe challenge. Quantum effects are furthermore important^91^,^92^ and have been proposed to be decisive in determining the difference in entropic contribution from HDL and LDL^11^. Including quantum effects adds additional cost to simulations, but recent developments in terms of coloured noise have reduced the expense of including this important effect^93^. An additional challenge to simulators is to predict or extract reliably experimental observables from the simulation where, in particular, techniques to reproduce the X-ray spectroscopies are currently being under strong development both within DFT^89^,^94^,^95^ and traditional quantum chemistry^96^,^97^.

## Additional information

**How to cite this article:** Nilsson, A. & Pettersson, L. G. M. The structural origin of anomalous properties of liquid water. *Nat. Commun.* 6:8998 doi: 10.1038/ncomms9998 (2015).

## A simplified picture of heterogeneous–homogeneous fluctuations

Fluctuations in a liquid could appear on various length and time scales. If the fluctuations are correlated over distances that extend over more than the molecular size, the fluctuations could give rise to different local structural regions that we denote patches. Here, in the figure below, we envision a heterogeneous picture with two local structural environments (blue and yellow), while structures representing a structural average in between these two extremes are indicated as green. The patches are not static but will rearrange into each other through structural fluctuations on some time-scale that is longer than the hydrogen bond lifetime. We also have the situation where the liquid only encompasses a homogeneous single structural environment with normal thermal fluctuations that is denoted a simple liquid.

In an illustrative picture we can liken the dynamics of the fluctuations in the heterogeneous case to that of a pendulum moving between two turning points. The speed is zero at the turning points and maximum at the point in the middle. Here the black curve shows such a scenario as time spent on various points along the fluctuating trajectory where the molecules of the liquid will spend most of the time at the extreme points and little time as they are converting in between. The timescales for the collective motion is expected to be a minimum of a few picoseconds but varying with temperature. This leads to an extreme heterogeneous situation where two well-defined structural classes develop on a temperature-dependent length and time scale. The length scale should correspond as a minimum to a factor 2 to 3 of the molecular dimension allowing for the existence of several shells. The boundary in between contains few molecules, as the time spent in this region is very low.

As the fluctuating trajectory becomes less extreme the molecules spend less time at the turning points and more time in the intermediate structures, and thereby the liquid becomes less heterogeneous. The patches have no time to develop and will eventually be on a molecular length scale. We denote this the ideal-mixture regime where the local structure becomes dominated by the intermediate structures. Here we consider this as a homogeneous structure with a very broad range of structures. In the final case of a homogeneous simple liquid there are thermal fluctuations around structures centred on the local structure dominating the respective end point. We will demonstrate that most probably these three situations are representative of water in different regions of the phase diagram.

## References

[b1] Ball P. H_2_O: A Biography of Water Weidenfeld & Nicolson (1999).

[b2] Debenedetti P. G. Supercooled and glassy water. J. Phys. Condens. Matter 15, R1669–R1726 (2003).

[b3] Kell G. S. Isothermal compressibility of liquid water at 1 atm. J. Chem. Eng. Data 15, 119–122 (1970).

[b4] Speedy R. J. & Angell C. A. Isothermal compressibility of supercooled water and evidence for a thermodynamic singularity at −45 °C. J. Chem. Phys. 65, 851–858 (1976).) ***This paper presents experimental evidence of a divergence of thermodynamic response functions at a temperature around 228 K in the supercooled regime*** .

[b5] Angell C. A., Sichina W. J. & Oguni M. Heat capacity of water at extremes of supercooling and superheating. J. Phys. Chem. 86, 998–1002 (1982).

[b6] Kumar P., Han S. & Stanley H. E. Anomalies of water and hydrogen bond dynamics in hydrophobic nanoconfinement. J. Phys. Condens. Matter 21, 504108 (2009). 21836219 10.1088/0953-8984/21/50/504108

[b7] Mishima O. & Stanley H. E. The relationship between liquid, supercooled and glassy water. Nature 396, 329–335 (1998).

[b8] Debenedetti P. G. & Stillinger F. H. Supercooled liquids and the glass transition. Nature 410, 259–267 (2001). 11258381 10.1038/35065704

[b9] Kell G. S. Density, thermal expansivity, and compressibility of liquid water from from 0° to 150 °C: correlations and tables for atmospheric pressure and saturation reviewed and expressed on 1968 temperature scale. J. Chem. Eng. Data 20, 97–105 (1975).

[b10] Zheleznyi B. V. The density of supercooled water. Russ. J. Phys. Chem. 43, 1311–1311 (1969).

[b11] Nilsson A., Huang C. & Pettersson L. G. M. Fluctuations in ambient water. J. Mol. Liq. 176, 2–16 (2012).

[b12] Nilsson A. & Pettersson L. G. M. Perspective on the structure of liquid water. Chem. Phys. 389, 1–34 (2011).

[b13] Kühne T. D. & Khaliullin R. Z. Electronic signature of the instantaneous asymmetry in the first coordination shell in liquid water. Nat. Commun. 4, 1450 (2013).) ***Here the authors demonstrate in simulations that asymmetrical structures evolve as an electronic structure effect that can be linked to previous observations with X-ray absorption spectroscopy*** . 23385594 10.1038/ncomms2459

[b14] Leetmaa M. *et al.* Diffraction and IR/Raman data do not prove tetrahedral water. J. Chem. Phys. 129, 084502 (2008). 19044830 10.1063/1.2968550

[b15] Russo J. & Tanaka H. Understanding water's anomalies with locally favoured structures. Nat. Commun. 5, 3556 (2014).) ***This paper presents a temperature-dependent two-state model that can explain both structural and thermodynamic properties of water*** . 24694550 10.1038/ncomms4556

[b16] Huang C. *et al.* The inhomogeneous structure of water at ambient conditions. Proc. Natl Acad. Sci. USA 106, 15214–15218 (2009).) ***This paper brings forward both spectroscopic and scattering evidence that water is inhomogeneous in its instantaneous structure at ambient conditions and proposes a unified picture of the ambient and supercooled regimes*** . 19706484 10.1073/pnas.0904743106 PMC2741230

[b17] Soper A. K. & Ricci M. A. Structures of high-density and low-density water. Phys. Rev. Lett. 84, 2881–2884 (2000).) ***This paper uses trends in neutron scattering experiments at various temperatures and pressures to extract pair-distribution functions for potential pure phases of HDL and LDL*** . 11018966 10.1103/PhysRevLett.84.2881

[b18] Bellissent-Funel M. C. Is there a liquid-liquid phase transition in supercooled water? Europhys. Lett. 42, 161–166 (1998).

[b19] Poole P. H., Sciortino F., Essmann U. & Stanley H. E. Phase-behavior of metastable water. Nature 360, 324–328 (1992).) ***As an explanation for the divergent thermodynamic response functions this paper proposes that a 2nd critical point and a liquid-liquid transition could exist in supercooled water*** .

[b20] Palmer J. C. *et al.* Metastable liquid-liquid transition in a molecular model of water. Nature 385–388 (2014).) ***Here the authors demonstrate that the ST2 model supports HDL and LDL coexistence as metastable phases with a free energy barrier towards ice crystallization*** . 24943954 10.1038/nature13405

[b21] Sellberg J. A. *et al.* Comparison of X-ray absorption spectra between water and ice: new ice data with low pre-edge absorption cross-section. J. Chem. Phys. 141, 034507 (2014). 25053326 10.1063/1.4890035

[b22] Nilsson A. *et al.* X-ray absorption spectroscopy and X-ray Raman scattering of water; an experimental view. J. El. Spec. Rel. Phen 177, 99–129 (2010).

[b23] Chen W., Wu X. & Car R. X-ray absorption signatures of the molecular environment in water and ice. Phys. Rev. Lett. 105, 017802 (2010). 20867480 10.1103/PhysRevLett.105.017802

[b24] Nordlund D. *et al.* Sensitivity of X-ray absorption spectroscopy to hydrogen bond topology. Phys. Rev. B 80, 233404 (2009).

[b25] Wernet P. *et al.* The structure of the first coordination shell in liquid water. Science 304, 995–999 (2004). 15060287 10.1126/science.1096205

[b26] Tse J. S. *et al.* X-ray Raman spectroscopic study of water in the condensed phases. Phys. Rev. Lett. 100, 095502 (2008). 18352721 10.1103/PhysRevLett.100.095502

[b27] Pylkkänen T. *et al.* Role of non-hydrogen-bonded molecules in the oxygen K-edge spectrum in ice. J. Phys. Chem. B 114, 3804–3808 (2010). 20187617 10.1021/jp912208v

[b28] Waluyo I., Nordlund D., Bergmann U., Pettersson L. G. M. & Nilsson A. A different view of structure-making and structure-breaking in alkali halide aqueous solutions through X-ray absorption spectroscopy. J. Chem. Phys. 140, 244506 (2014). 24985653 10.1063/1.4881600

[b29] Leberman R. & Soper A. K. Effect of high-salt concentrations on water-structure. Nature 378, 364–366 (1995). 18286746 10.1038/378364a0

[b30] Fuchs O. *et al.* Isotope and temperature effects in liquid water probed by X-ray absorption and resonant X-ray emission spectroscopy. Phys. Rev. Lett. 100, 027801 (2008). 18232928 10.1103/PhysRevLett.100.027801

[b31] Tokushima T. *et al.* High resolution X-ray emission spectroscopy of liquid water: the observation of two structural motifs. Chem. Phys. Lett. 460, 387–400 (2008).

[b32] Yin Z. *et al.* Probing the Hofmeister effect with ultrafast core-hole spectroscopy. J. Phys. Chem. B 118, 9398–9403 (2014). 25029209 10.1021/jp504577a

[b33] Nilsson A. *et al.* Resonant inelastic X-ray scattering of water. J. El. Spec. Rel. Phen. 188, 84–100 (2013).

[b34] Tokushima T. *et al.* High resolution X-ray emission spectroscopy of water and its assignment based on two structural motifs. J. El. Spec. Rel. Phen. 177, 192–205 (2010).

[b35] Fuchs O. *et al.* Reply to comment on “Isotope and temperature effects in liquid water probed by X-ray absorption and resonant X-Ray emission spectroscopy”. Phys. Rev. Lett. 100, 249802 (2008). 10.1103/PhysRevLett.100.027801 18232928

[b36] Pettersson L. G. M. *et al.* Comment on “Isotope and temperature effects in liquid water probed by X-ray absorption and resonant X-ray emission spectroscopy”. Phys. Rev. Lett. 100, 249801 (2008). 18643637 10.1103/PhysRevLett.100.249801

[b37] Holten V. & Anisimov M. A. Entropy-driven liquid–liquid separation in supercooled water. Sci. Rep. 2, 713 (2012).) ***Here the authors show that a two-state model can be used to quantitatively describe all thermodynamic response functions and predict the location of a 2nd critical point*** . 23056905 10.1038/srep00713 PMC3465811

[b38] Sun Q. Raman spectroscopic study of the effects of dissolved NaCl on water structure. Vib. Spectrosc. 62, 110–114 (2012).

[b39] Sun Q. Local statistical interpretation for water structure. Chem. Phys. Lett. 568–569, 90–94 (2013).

[b40] Taschin A., Bartolini P., Eramo R., Righini R. & Torre R. Evidence of two distinct local structures of water from ambient to supercooled conditions. Nat. Commun. 4, 2401 (2013).) ***This paper shows, based on time-dependent optical Kerr measurements, that vibrational frequencies in the low-energy spectral regime of water can be interpreted as supporting a two-state model*** . 24029922 10.1038/ncomms3401

[b41] Bakker H. J. & Skinner J. L. Vibrational spectroscopy as a probe of structure and dynamics in liquid water. Chem. Rev. 110, 1498–1517 (2010). 19916491 10.1021/cr9001879

[b42] Nibbering E. T. J. & Elsaesser T. Ultrafast vibrational dynamics of hydrogen bonds in the condensed phase. Chem. Rev. 104, 1887–1914 (2004). 15080715 10.1021/cr020694p

[b43] Heyden M. *et al.* Dissecting the THz spectrum of liquid water from first principles via correlations in time and space. Proc. Natl Acad. Sci. USA 107, 12068–12073 (2010). 20566886 10.1073/pnas.0914885107 PMC2901429

[b44] Nagata Y., Yoshimune S., Hsieh C. S., Hunger J. & Bonn M. Ultrafast vibrational dynamics of water disentangled by reverse nonequilibrium ab initio molecular dynamics simulations. Phys. Rev. X 5, 021002 (2015).

[b45] Walrafen G. E. Raman spectral studies of water structure. J. Chem. Phys. 40, 3249–3256 (1964).) ***This paper was the early Raman spectroscopic experimental work on water that generated more than half a century of follow-up studies*** .

[b46] Minceva-Sukarova B., Sherman W. F. & Wilkinson G. R. The Raman spectra of ice (I_h_, II, III, V, VI, and IX) as functions of pressure and temperature. J. Phys. C Solid State Phys. 17, 5833–5850 (1984).

[b47] van der Post S. T. *et al.* Strong frequency dependence of vibrational relaxation in bulk and surface water reveals sub-picosecond structural heterogeneity. Nat. Commun. 6, 8384 (2015). 26382651 10.1038/ncomms9384 PMC4595750

[b48] Harada Y. *et al.* Selective probing of the OH or OD stretch vibration in liquid water using resonant inelastic soft-X-ray scattering. Phys. Rev. Lett. 111, 193001 (2013). 24266469 10.1103/PhysRevLett.111.193001

[b49] Skinner L. B., Benmore C. J., Neuefeind J. C. & Parise J. B. The structure of water around the compressibility minimum. J. Chem. Phys. 141, 214507 (2014).) ***This paper shows tremendous advances in temperature-dependent X-ray scattering measurements of water where fine details on long-range correlations can be extracted*** . 25481152 10.1063/1.4902412

[b50] Khaliullin R. Z. & Kühne T. D. The nature of the asymmetry in the hydrogen-bond networks of hexagonal ice and liquid water. J. Am. Chem. Soc. 136, 3395–3399 (2014). 24521433 10.1021/ja411161a

[b51] Abascal J. L. F. & Vega C. A general purpose model for the condensed phases of water: TIP4P/2005. J. Chem. Phys. 123, 234505 (2005). 16392929 10.1063/1.2121687

[b52] Shiratani E. & Sasai M. Growth and collapse of structural patterns in the hydrogen bond network in liquid water. J. Chem. Phys. 104, 7671–7680 (1996).

[b53] Huang C. *et al.* Wide-angle X-ray diffraction and molecular dynamics study of medium-range order in ambient and hot water. Phys. Chem. Chem. Phys. 13, 19997–20007 (2011). 22009343 10.1039/c1cp22804h

[b54] Santra B., DiStasio R. A. Jr, Martelli F. & Car R. Local structure analysis in *ab initio* liquid water. Mol. Phys. 113, 2829–2841 (2015).

[b55] Huang C. *et al.* Increasing correlation length in bulk supercooled H_2_O, D_2_O and NaCl solution determined from small angle X-ray scattering. J. Chem. Phys. 133, 134504 (2010). 20942543 10.1063/1.3495974 PMC2966487

[b56] Skinner L. B. *et al.* Benchmark oxygen-oxygen pair-distribution function of ambient water from X-ray diffraction measurements with a wide Q-range. J. Chem. Phys. 138, 074506 (2013). 23445023 10.1063/1.4790861

[b57] Overduin S. D. & Patey G. N. Understanding the structure factor and isothermal Compressibility of ambient water in terms of local structural environments. J. Phys. Chem. B 116, 12014–12020 (2012). 22963671 10.1021/jp3075749

[b58] Clark G. N. I., Cappa C. D., Smith J. D., Saykally R. J. & Head-Gordon T. The structure of ambient water. Mol. Phys. 108, 1415–1433 (2010).

[b59] Clark G. N. I., Hura G., Teixeira J., Soper A. K. & Head-Gordon T. Small-angle scattering and the structure of ambient liquid water. Proc. Natl Acad. Sci. USA 107, 14003–14007 (2010). 20647388 10.1073/pnas.1006599107 PMC2922569

[b60] Soper A. K. Recent water myths. Pure Appl. Chem. 82, 1855–1867 (2010).

[b61] Wikfeldt K. T., Huang C., Nilsson A. & Pettersson L. G. M. Enhanced small-angle scattering connected to the Widom line in simulations of supercooled water. J. Chem. Phys. 134, 214506 (2011). 21663366 10.1063/1.3594545

[b62] Wikfeldt K. T., Nilsson A. & Pettersson L. G. M. Spatially inhomogeneous bimodal inherent structure in simulated liquid water. Phys. Chem. Chem. Phys. 13, 19918–19924 (2011). 21915406 10.1039/c1cp22076d

[b63] Stokely K., Mazza M. G., Stanley H. E. & Franzese G. Effect of hydrogen bond cooperativity on the behavior of water. Proc. Natl Acad. Sci. USA 107, 1301–1306 (2010). 20080604 10.1073/pnas.0912756107 PMC2824356

[b64] Tulk C. A. *et al.* Structural studies of several distinct metastable forms of amorphous ice. Science 297, 1320–1323 (2002). 12193779 10.1126/science.1074178

[b65] Kesselring T. A., Franzese G., Buldyrev S. V., Herrmann H. J. & Stanley H. E. Nanoscale dynamics of phase flipping in water near its hypothesized liquid-liquid critical point. Sci. Rep. 2, 474 (2012). 22761987 10.1038/srep00474 PMC3386518

[b66] Limmer D. T. & Chandler D. The putative liquid-liquid transition is a liquid-solid transition in atomistic models of water. J. Chem. Phys. 135, 134503 (2011). 21992320 10.1063/1.3643333

[b67] Limmer D. T. & Chandler D. The putative liquid-liquid transition is a liquid-solid transition in atomistic models of water. II. J. Chem. Phys. 138, 214504 (2013). 23758385 10.1063/1.4807479

[b68] Stillinger F. H. & Rahman A. Improved simulation of liquid water by molecular dynamics. J. Chem. Phys. 60, 1545–1557 (1974).

[b69] Smallenburg F. & Scortino F. Tuning the liquid-liquid transition by modulating the hydrogen bond angular flexibility in a model for water. Phys. Rev. Lett. 115, 015701 (2015). 26182107 10.1103/PhysRevLett.115.015701

[b70] Holten V., Palmer J. C., Poole P. H., Debenedetti P. G. & Anisimov M. A. Two-state thermodynamics of the ST2 model for supercooled water. J. Chem. Phys. 140, 104502 (2014). 24628177 10.1063/1.4867287

[b71] Sellberg J. A. *et al.* Ultrafast X-ray probing of water structure below the homogeneous ice nucleation temperature. Nature 510, 381–384 (2014).) ***This paper demonstrates how the “No-man's land” region can be investigated through experiments based on fast cooling and ultrafast probing using an X-ray laser*** . 24943953 10.1038/nature13266

[b72] Mason B. J. The supercooling and nucleation of water. Adv. Phys. 7, 221–234 (1958).

[b73] Angell C. A. Supercooled water: two phases? Nat. Mater. 13, 673–675 (2014). 24947781 10.1038/nmat4022

[b74] Mishima O. Volume of supercooled water under pressure and the liquid-liquid critical point. J. Chem. Phys. 133, 144503 (2010). 20950013 10.1063/1.3487999

[b75] Mishima O. & Stanley H. E. Decompression-induced melting of ice IV and the liquid-liquid transition in water. Nature 392, 164–168 (1998).

[b76] Limmer D. T. & Chandler D. Time scales of supercooled water and implications for reversible polyamorphism. Mol. Phys. 113, 2799–2804 (2015).

[b77] Xu L. *et al.* Relation between the Widom line and the dynamic crossover in systems with a liquid–liquid phase transition. Proc. Natl Acad. Sci. USA 102, 16558–16562 (2005).) ***Here the concept of Widom line is presented as the loci of maxima in the thermodynamic response functions extending from the phase coexistence line into the one-phase region*** . 16267132 10.1073/pnas.0507870102 PMC1283834

[b78] Bosio L., Teixeira J. & Bellissent-Funel M. C. Enhanced density fluctuations in water analyzed by neutron scattering. Phys. Rev. A 39, 6612–6613 (1989). 9901270 10.1103/physreva.39.6612

[b79] Gallo P., Corradini D. & Rovere M. Widom line and dynamical crossovers as routes to understand supercritical water. Nat. Commun. 5, 5806 (2014). 25512253 10.1038/ncomms6806

[b80] Appignanesi G. A., Rodriguez Friz J. A. & Sciortino F. Evidence of two-state picture for supercooled water and its connections to glassy dynamics. Eur. Phys. J. E Soft Matter 29, 305–310 (2009). 19603209 10.1140/epje/i2009-10478-6

[b81] Pi H. L. *et al.* Anomalies in water as obtained from computer simulations of the TIP4P/2005 model: density maxima, and density, isothermal compressibility and heat capacity minima. Mol. Phys. 107, 365–374 (2009).

[b82] Amann-Winkel K. *et al.* Water's second glass transition. Proc. Natl Acad. Sci. USA 110, 17720–17725 (2013).) ***This paper demonstrates that there is a 2nd glass transition which could be evidence of two liquid phases in the regime close to the temperature where the amorphous phases are metastable*** . 24101518 10.1073/pnas.1311718110 PMC3816484

[b83] Fecko C. J., Eaves J. D., Loparo J. J., Tokmakoff A. & Geissler P. L. Ultrafast hydrogen-bond dynamics in the infrared spectroscopy of water. Science 301, 1698–1702 (2003). 14500975 10.1126/science.1087251

[b84] Garret-Roe S., Perakis F., Rao F. & Hamm P. Three-dimensional infrared spectroscopy of isotope-substituted liquid water reveals heterogeneous dynamics. J. Phys Chem. B 115, 6976–6984 (2011). 21561076 10.1021/jp201989s

[b85] Ruocco G. & Sette F. The history of the “fast sound” in liquid water. Condens. Matter Phys. 11, 29–46 (2008).

[b86] Laksmono H. *et al.* Anomalous behavior of the homogeneous ice nucleation rate in “no-man's land”. J. Phys. Chem. Lett. 6, 2826–2832 (2015). 26207172 10.1021/acs.jpclett.5b01164 PMC4507474

[b87] Wang Y., Huang X., Shepler B. C., Braams B. J. & Bowman J. M. Flexible, ab initio potential, and dipole moment surfaces for water. I. Tests and applications for clusters up to the 22-mer. J. Chem. Phys. 134, 094509 (2011). 21384987 10.1063/1.3554905

[b88] Del Ben M., Schönherr M., Hutter J. & VandeVondele J. Bulk liquid water at ambient temperature and pressure from MP2 theory. J. Phys. Chem. Lett. 4, 3753–3759 (2013). 10.1021/jz501672u 26278261

[b89] Kong L., Wu X. & Car R. Roles of quantum nuclei and inhomogeneous screening in the X-ray absorption spectra of water and ice. Phys. Rev. B 86, 134203 (2012).

[b90] Wan Q., Spanu L., Galli G. A. & Gygi F. Raman spectra of liquid water from *ab initio* molecular dynamics: vibrational signatures of charge fluctuations in the hydrogen bond network. J. Chem. Theory Comp. 9, 4124–4130 (2013). 10.1021/ct4005307 26592405

[b91] Marx D., Tuckerman M. E., Hutter J. & Parrinello M. The nature of the hydrated excess proton in water. Nature 397, 601–604 (1999).

[b92] Habershon S., Markland T. E. & Manolopolous D. E. Competing quantum effects in the dynamics of a flexible water model. J. Chem. Phys. 131, 024501 (2009). 19603998 10.1063/1.3167790

[b93] Ceriotti M., Bussi G. & Parrinello M. Langevin equation with colored noise for constant-temperature molecular dynamics simulations. Phys. Rev. Lett. 102, 020601 (2009). 19257259 10.1103/PhysRevLett.102.020601

[b94] Vinson J., Kas J. J., Rehr J. J., Vila F. D. & Shirley E. L. Theoretical optical and X-ray spectra of liquid and solid H_2_O. Phys. Rev. B 85, 045101 (2012).

[b95] Besley N. A., Peach M. J. G. & Tozer D. J. Time-dependent density functional theory calculations of near-edge X-ray absorption fine structure with short-range corrected functionals. Phys. Chem. Chem. Phys. 11, 10350–10358 (2009). 19890519 10.1039/b912718f

[b96] Ekström U., Norman P., Carravetta V. & Ågren H. Polarization propagator for X-ray spectra. Phys. Rev. Lett. 97, 143001 (2006). 17155244 10.1103/PhysRevLett.97.143001

[b97] Nilsson A., Schlesinger D. & Pettersson L. G. M. in Proceedings of the International School of Physics "Enrico Fermi" eds Debenedetti P. G., Ricci M. A., Bruni F.) Vol. 187, (IOS Press (2015).

